# Two-sample Mendelian randomization analysis evaluates causal associations between inflammatory bowel disease and osteoporosis

**DOI:** 10.3389/fpubh.2023.1151837

**Published:** 2023-05-26

**Authors:** Zhujiang Dai, Weimin Xu, Rui Ding, Xiang Peng, Xia Shen, Jinglue Song, Peng Du, Zhongchuan Wang, Yun Liu

**Affiliations:** ^1^Department of Colorectal Surgery, Xinhua Hospital, Shanghai Jiaotong University School of Medicine, Shanghai, China; ^2^Shanghai Colorectal Cancer Research Center, Shanghai, China

**Keywords:** inflammatory bowel disease, osteoporosis, Mendelian randomization, causal association, GWAS

## Abstract

**Introduction:**

Over the past few years, multiple observational studies have speculated a potential association between inflammatory bowel disease (IBD), which includes ulcerative colitis (UC) and Crohn’s disease (CD), and osteoporosis. However, no consensus has been reached regarding their interdependence and pathogenesis. Herein, we sought to further explore the causal associations between them.

**Methods:**

We validated the association between IBD and reduced bone mineral density in humans based on genome-wide association studies (GWAS) data. To investigate the causal relationship between IBD and osteoporosis, we performed a two-sample Mendelian randomization study using training and validation sets. Genetic variation data for IBD, CD, UC, and osteoporosis were derived from published genome-wide association studies in individuals of European ancestry. After a series of robust quality control steps, we included eligible instrumental variables (SNPs) significantly associated with exposure (IBD/CD/UC). We adopted five algorithms, including MR Egger, Weighted median, Inverse variance weighted, Simple mode, and Weighted mode, to infer the causal association between IBD and osteoporosis. In addition, we evaluated the robustness of Mendelian randomization analysis by heterogeneity test, pleiotropy test, leave-one-out sensitivity test, and multivariate Mendelian randomization.

**Results:**

Genetically predicted CD was positively associated with osteoporosis risk, with ORs of 1.060 (95% CIs 1.016, 1.106; *p* = 0.007) and 1.044 (95% CIs 1.002, 1.088; *p* = 0.039) for CD in the training and validation sets, respectively. However, Mendelian randomization analysis did not reveal a significant causal relationship between UC and osteoporosis (*p* > 0.05). Furthermore, we found that overall IBD was associated with osteoporosis prediction, with ORs of 1.050 (95% CIs 0.999, 1.103; *p* = 0.055) and 1.063 (95% CIs 1.019, 1.109; *p* = 0.005) in the training and validation sets, respectively.

**Conclusion:**

We demonstrated the causal association between CD and osteoporosis, complementing the framework for genetic variants that predispose to autoimmune disease.

## Introduction

With an overall increase in human lifespan, aging-related diseases are becoming increasingly prevalent each year (1, 2). Among them, osteoporosis is a common systemic skeletal disease characterized by an imbalance in bone formation and resorption (3). Currently, the gold standard for diagnosing osteoporosis is through bone mineral density (BMD) measurements of the lumbar spine, proximal femur, and distal forearm, using dual-energy X-ray bone absorptiometry (DXA) (4). It is widely believed that age, hormones (glucocorticoids, steroid hormones), nutrition (coffee, alcohol, vitamin D, calcium), and autochthonous diseases (inflammatory diseases, gastrointestinal diseases, blood disorders) are associated with reduced bone density and increased risk of osteoporosis (5). Therefore, it is essential to identify osteoporosis’s causes and associated risk factors.

Several gastrointestinal disorders have been associated with osteoporosis and bone loss, including inflammatory bowel disease, celiac disease, and chronic liver disease (6, 7). Inflammatory bowel disease (IBD) is an idiopathic disease that can be categorized into two major types: Crohn’s disease and ulcerative colitis. IBD’s pathogenesis is thought to stem from the interplay of genetic susceptibility and other risk factors (8). The incidence of osteoporosis and the associated risk of pathological fracture in patients with IBD has increased over the years, potentially caused by a loss of bone minerals due to IBD, an imbalance between osteoblasts and osteoclasts triggered by inflammatory mediators, and the administration of glucocorticoids (9–11). However, establishing a causal association between IBD and osteoporosis from an epidemiological perspective has been challenging, with factors such as gut microbiota, nutritional intake, and hormonal interventions complicating the issue (12, 13). Besides, bone densitometry is relatively rare and not commonly available in general community hospitals. Moreover, clinicians do not generally provide precise guidance on the frequency of DXA scans for IBD patients. Due to differences in study design and ethnicity, a clear causal association between IBD and osteoporosis remains ambiguous.

Mendelian randomization (MR) uses genetic variation to assess causal relationships between exposures and outcomes, similar to randomized controlled studies (14). By including genetically assigned variation obtained through meiosis as an exposure-related instrumental variable (IV), the analysis results are less affected by confounding factors. To determine whether a causal relationship exists between IBD and osteoporosis, we conducted a two-sample MR analysis using publicly accessible genome-wide association studies (GWAS) datasets.

## Materials and methods

### Data sources and study design

**Figure 1:**
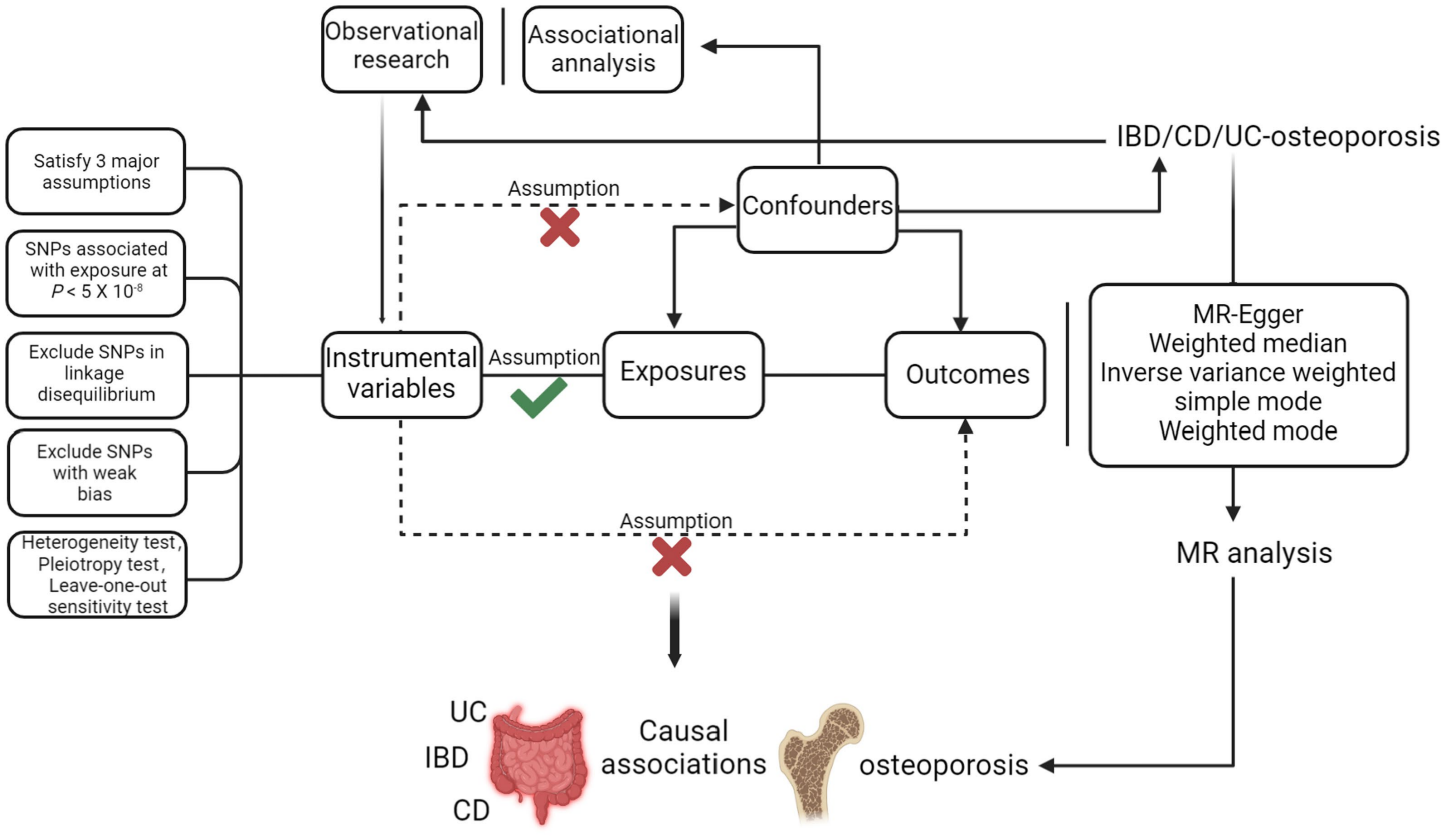
Procedure for an MR analysis of causal associations between IBD (including UC/CD) and osteoporosis. Checks indicate a correlation between IVs and exposure, while crosses suggest no correlation with confounders or outcomes.

The training set was derived from the most extensive genome-wide association study (GWAS, https://gwas.mrcieu.ac.uk/), where cohorts included IBD (*N* = 12,882 cases, 21,770 controls; SNPs = 12,716,084), UC (*N* = 6,968 cases, 20,464 controls; SNPs = 12,255,197), CD (*N* = 5,956 cases,14,927 controls; SNPs = 12,276,506) (14). The GWAS data used for validation was from the United Kingdom IBD Genetics Consortium (UKIBDGC) and the publicly available International Inflammatory Bowel Disease Genetics Consortium (IIBDGC) pooled studies, including IBD (*N* = 25,042 cases, 34,915 controls; SNPs = 9,619,016), UC (*N* = 12,366 cases, 33,609 controls; SNPs = 9,474,559), CD (*N* = 12,194 cases, 28,072 controls; SNPs = 9,457,998) (15). These include the UK low coverage whole genome sequencing IBD study, the UK HumanCoreExome genotyped IBD study, and the IIBDGC genotyped IBD (including CD and UC) study. The data originates from European populations and has undergone strict quality control to eliminate potential overlap. The osteoporosis cohort included 3,203 cases and 209,575 controls, comprising 16,380,452 SNPs. Population selection was based on the International Classification of Diseases-10 (ICD-10). Currently, given the lack of a gold standard for diagnosing IBD, clinical investigators rely on several factors, including the patient’s clinical presentation, standard diagnoses like endoscopy and histopathology, and excluding infectious and non-infectious colitis. Diagnostic criteria for UC include persistent or recurrent diarrhoea, mucus stools, bloody stools with abdominal pain, and varying degrees of systemic symptoms. Endoscopic examination may reveal diffuse mucosal involvement, loss of vascular texture and mucosal roughness, and multiple vesicles or pseudo-ulcers. Pathological examination during the active phase may show diffuse inflammatory cell infiltration in the mucosal cavity, crypt abscesses, and cupulocytopenia. Diagnostic criteria for CD include similar clinical presentations and endoscopic examination may reveal discontinuous lesions with the mucosa between the lesions completely normal, as well as oval signs and varying degrees of intestinal wall thickening. Pathological examination may show focal chronic inflammation, focal structural abnormalities of the crypt, and non-caseating granulomas. To diagnose osteoporosis, a DXA examination at the femoral neck and lumbar spine is considered the gold standard. All datasets provided in GWAS have been approved by the relevant ethics committees. The data derivation process of this study is shown in Figure 1.

### Instrumental variables selection and quality control

In MR, genetic variants are used as IVs to assess the causal effect of exposure on outcomes (16). The fundamental requirements for genetic variation to meet the criteria for instrumental variables were: (1) genetic variation is associated with exposure; (2) genetic variation is not associated with confounders affecting exposure/outcome; (3) genetic variation may only be associated with outcome through exposure. Prior to MR analysis, we performed multi-step quality control of SNPs to ensure that qualified IVs were obtained. First, we ensured that all IVs were not in linkage disequilibrium to avoid bias in the results due to strong linkage disequilibrium. To effectively avoid potential linkage disequilibrium interference between SNPs, the following screening criteria were used to identify SNPs (17): (1) Significant threshold associated with IV: *p* < 5 × 10^−8^; (2) r^2^ = 0.001; (3) The physical distance between genes kb = 10,000.

### Statistical analyses

MR analysis was based on genetic variants as IVs to estimate the causal association of exposure on outcome. We combined multiple independent data (beta coefficients and standard errors) and adopted different algorithms to assess causal associations between IBD (including UC/CD) and osteoporosis. We adopted five algorithms of MR Egger (18), Weighted median (19), Inverse variance weighted (IVW) (20), Simple mode, and Weighted mode to estimate the causal association between IBD and osteoporosis. All analyses were adjusted *via* the Bonferroni method (21).

### Correlation test of IVs and exposure

To ensure that IVs and exposure were sufficiently correlated, we incorporated the F-score to prevent any biases from weak IVs (17). Typically, an F-score greater than 10 indicates a lack of weak IV bias. The formulas for calculating the F score and *R*^2^ are as follows. “*n*” represents the sample size, and “*k*” represents the number of SNPs included. The sample size of this study far exceeds the number of SNPs included, thus avoiding the possibility of weak IV bias.


$$F-\mathrm{Score}=\frac{R^{2}\left(n-k-1\right)}{k\left(1-R^{2}\right)}\times 100\%$$

$$R^{2}={\left(\frac{\beta }{se\left(\beta \right)\times \sqrt{n}}\right)}^{2}$$

### Heterogeneity test

We used Cochran’s Q statistic to test for heterogeneity of the included IVs (22). A *p*-value <0.05 indicated significant heterogeneity. The heterogeneity results are shown in Table 1.

**Table 1:** Significant MR estimates from IBD, CD, and UC on genetically predicted osteoporosis.

Category	Exposure	Outcome	Cochran’s Q derived *p* value	Egger intercept	MR-egger intercept derived *p* value	MR-PRESSO global test derived *p* value	*F* score
Training set	Inflammatory bowel disease	Osteoporosis	0.403	−0.001	0.948	0.371	62.378
	Crohn’s disease	Osteoporosis	0.487	−0.002	0.833	0.501	58.961
	Ulcerative colitis	Osteoporosis	0.562	−0.014	0.422	0.539	55.824
Validation set	Inflammatory bowel disease	Osteoporosis	0.477	0.007	0.171	0.458	70.034
	Crohn’s disease	Osteoporosis	0.690	0.013	0.178	0.705	80.293
	Ulcerative colitis	Osteoporosis	0.341	−0.027	0.076	0.347	64.576

### Pleiotropy test

We used the intercept term of MR Egger’s method to test whether there was horizontal pleiotropy in IVs (18, 23). A non-zero intercept term indicated the presence of horizontal pleiotropy. If the intercept term equaled 0 or was statistically insignificant, the MR Egger regression’s slope represented the estimated causal effect of exposure and outcome. In addition, we used the MR-PRESSO package (MR-PRESSO outlier test) to identify and remove any SNPs with significant differences to correct for horizontal pleiotropy (24). To avoid potential confounding, investigated possible associations of each instrument SNP with plausible confounders in the PhenoScanner GWAS database (*p* < 5 × 10^−8^). While all IVs were not associated with outcomes, residual confounding factors may still exist due to potential horizontal polymorphisms, despite our validation and culling efforts.

### Leave-one-out sensitivity test

After sequentially removing each IV using the leave-one-out sensitivity test, we performed IVW MR analysis on the remaining IVs (25). To determine the existence of abnormal IVs that significantly impact the causal effect estimates, we assessed the stability of the effect estimates.

### Ethics statement

Our study was based on publicly available GWAS pooled data, the included studies were approved by the institutional ethics review board, and all participants provided written informed consent.

### Statistics

All analyses were performed based on R(version 4.1.2) and the TwoSampleMR package. A *p*-value <0.05 was considered statistically significant.

## Results

The findings of our study were based on the radial IVW method, which employs adjusted second-order weights, while the other four methods are merely supplementary resources. Estimates from the MR analysis are detailed in Supplementary data.^1^

### Two-sample MR analysis for the causal association between IBD/CD/UC and osteoporosis

We identified 62, 31, and 51 robust single nucleotide polymorphisms (SNPs) as instrumental variables for IBD, UC, and CD, respectively, after excluding pleiotropic SNPs. The percentage of horizontal pleiotropic outliers ranged from 0 to 3.1%. Low heterogeneity was observed for all diseases using the IVW method, with Cochran’s Q-derived *p* values of 0.403 for IBD, 0.562 for UC, and 0.487 for CD. Results from MR Egger regression showed no bias due to genetic pleiotropy, with *p*-values ranging from 0.422 to 0.948. The MR-PRESSO global test revealed no weak IVs bias, with F-scores larger than 10 for all diseases and p-values ranging from 0.371 to 0.539.

**Figure 2:**
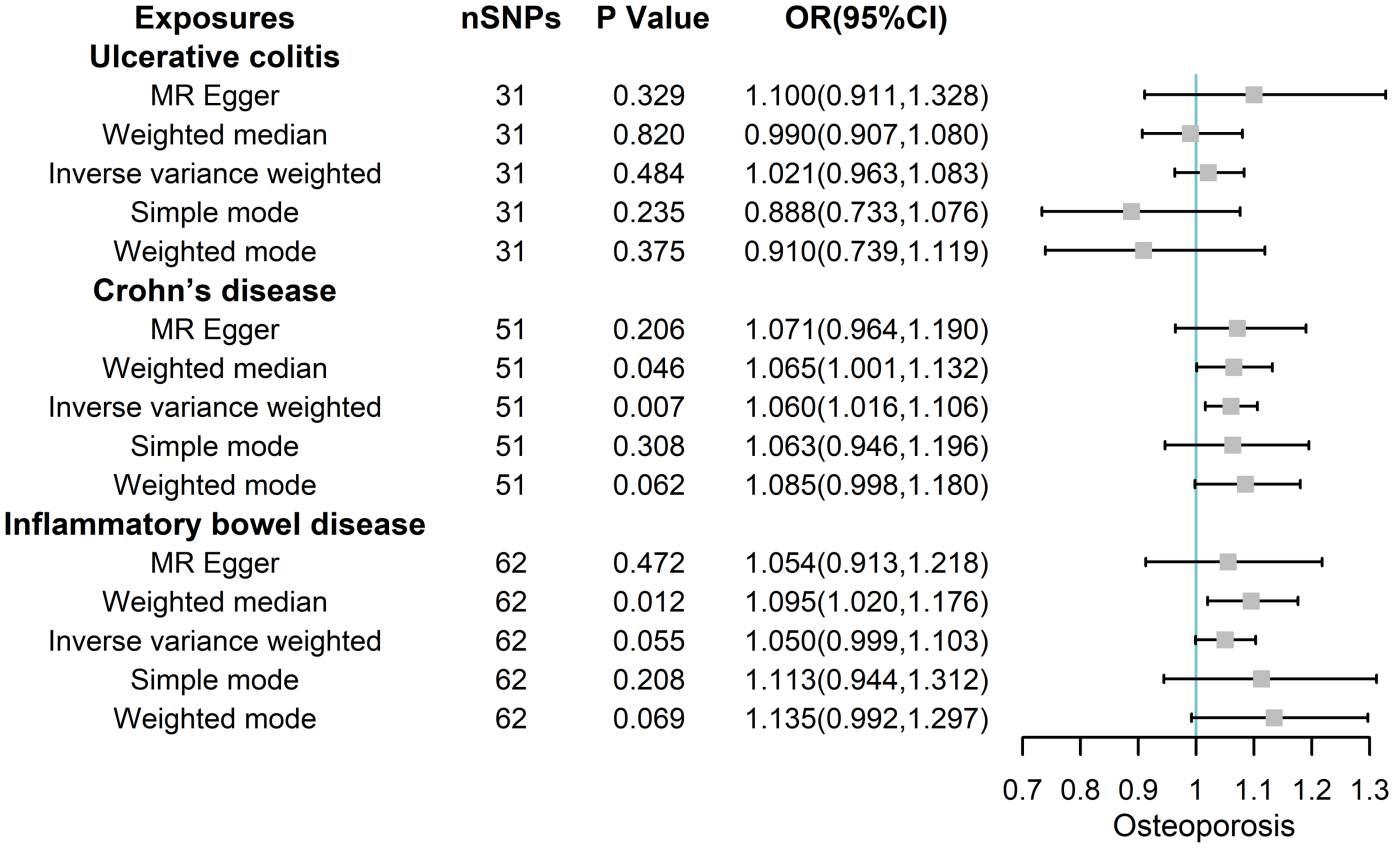
The risk association between IBD (including UC/CD) and osteoporosis in the training set visualized in a forest plot.

Therefore, these IVs were robust and feasible for assessing the causal association between IBD/CD/UC and osteoporosis. We evaluated the causal association between IBD/CD/UC and osteoporosis (mainly IVW) using five algorithms (MR Egger, Weighted median, IVW, Simple mode, and Weighted mode analysis), which yielded ORs of 1.054 (95% CIs 0.913, 1.218; *p* = 0.472), 1.095 (95% CIs 1.020, 1.176; *p* = 0.012),1.050 (95% CIs 0.999, 1.103; *p* = 0.055), 1.113 (95% CIs 0.944, 1.312; *p* = 0.208) and 1.135 (95% CIs 0.992, 1.297; *p* = 0.069) for IBD (Figure 2). The corresponding ORs for UC and osteoporosis were 1.100 (95% CIs 0.911, 1.328; *p* = 0.329), 0.990 (95% CIs 0.907, 1.080; *p* = 0.820),1.021 (95% CIs 0.963, 1.083; *p* = 0.484), 0.888 (95% CIs 0.733, 1.076; *p* = 0.235) and 0.910 (95% CIs 0.739, 1.119; *p* = 0.375). Finally, the corresponding ORs for CD and osteoporosis were 1.071 (95% CIs 0.964, 1.190; *p* = 0.206), 1.065 (95% CIs 1.001, 1.132; *p* = 0.046), 1.060 (95% CIs 1.016, 1.106; *p* = 0.007), 1.063 (95% CIs 0.946, 1.196; *p* = 0.308) and 1.085 (95% CIs 0.998, 1.180; *p* = 0.062), respectively. Collectively, these results illustrate that CD may increase the risk of osteoporosis from a genetic perspective.

**Figure 3:**
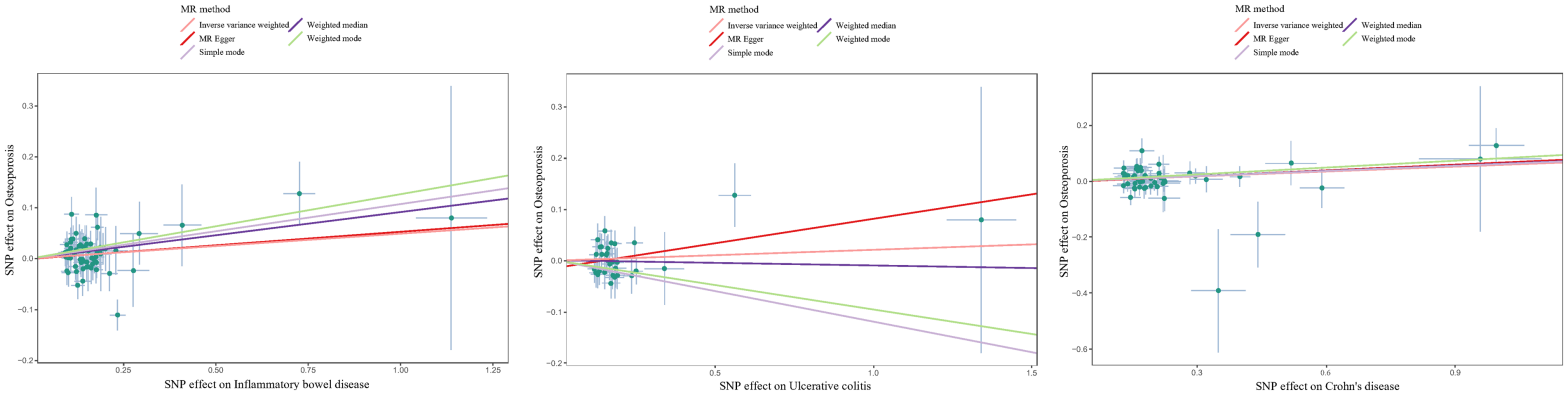
Scatter plots to estimate the genetic risk of osteoporosis in training set IBD (including UC/CD). SNP: Single Nucleotide Polymorphism. The slope of each line represents the effect estimation of different methods using MR.

The estimated effect sizes of SNPs on IBD/UC/CD and osteoporosis are shown in scatter plots. The selected IBD/CD/UC SNP effects (X-axis) and osteoporosis SNP effects (Y-axis) were plotted, and the results showed that there was a significant positive causal effect on the total effect of IBD/CD/UC and the occurrence of osteoporosis (Figure 3). However, the single SNP IVW analysis and the leave-one-out sensitivity analysis only suggested a causal effect of CD on osteoporosis(Supplementary Figures S1, S2). Taken together, there was a positive correlation between genetically predicted CD and osteoporosis.

We selected an IBD dataset with a larger sample size than the training set as the validation set. After removing pleiotropic SNPs, we obtained 102 SNPs for IBD, 48 for UC, and 80 for CD, all of which were robust instrumental variables. The percentages of horizontal polymorphic outliers for IBD/CD/UC were 10.5, 7.0, and 15.8%, respectively. The heterogeneity test by the IVW method showed that the heterogeneity among genes was low (IBD Cochran’s derived *p* value =0.477; UC Cochran’s Q derived p value =0.341; CD Cochran’s Q derived p value =0.690) (Table 1). The results of MR Egger regression suggested that genetic pleiotropy did not introduce any bias (IBD intercept = 0.007, *p* = 0.171; UC intercept = −0.027, *p* = 0.076; CD intercept = 0.013, *p* = 0.178). The MR-PRESSO global test yielded *p*-values of 0.458, 0.705, and 0.347 for IBD, CD, and UC, respectively, when tested against osteoporosis. Furthermore, the F-scores were well above 10, with values of 70.034, 64.576, and 80.293, indicating the absence of any weak IV bias.

**Figure 4:**
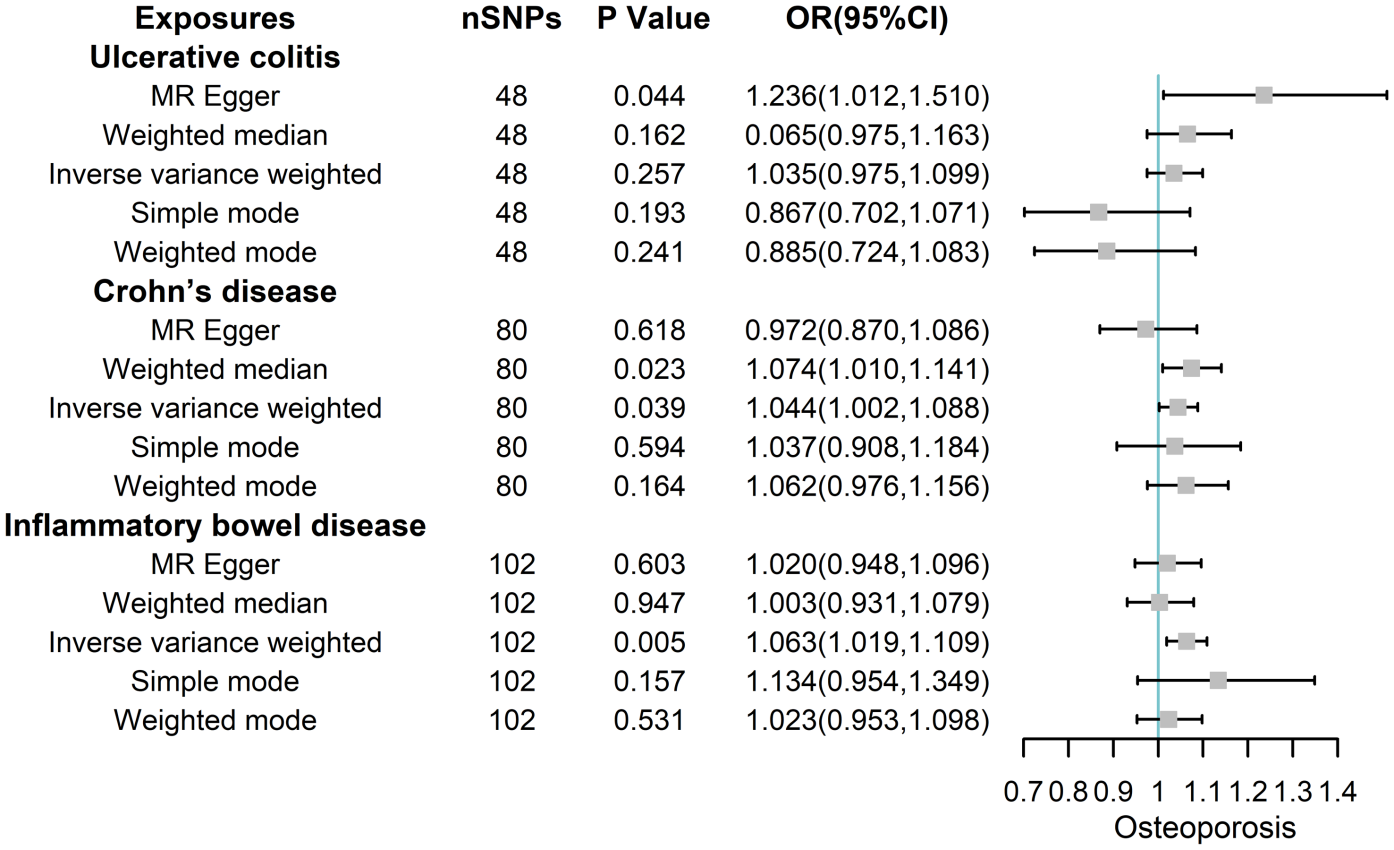
The risk association between IBD (including UC/CD) and osteoporosis in the validation set visualized in a forest plot.

MR Egger, Weighted median, IVW, Simple mode, and Weighted mode analysis were used to calculate the ORs of IBD, yielding ORs of 1.020 (95% CIs 0.948, 1.096; *p* = 0.603), 1.003 (95% CIs 0.931, 1.079; *p* = 0.947), 1.063 (95% CIs 1.019, 1.109; *p* = 0.005), 1.134 (95% CIs 0.954, 1.349; *p* = 0.157), and 1.023 (95% CIs 0.953, 1.098; *p* = 0.531) as shown in Figure 4. The corresponding ORs of UC with osteoporosis were 1.236 (95% CIs 1.012, 1.510; *p* = 0.044), 0.065 (95% CIs 0.975, 1.163; *p* = 0.162), 1.035 (95% CIs 0.975, 1.099; *p* = 0.257), 0.867 (95% CIs 0.702, 1.071; *p* = 0.193) and 0.885 (95% CIs 0.724, 1.083; *p* = 0.241). The corresponding ORs of CD with osteoporosis were 0.972 (95% CIs 0.870, 1.086; *p* = 0.618), 1.074(95% CIs 1.010, 1.141; *p* = 0.023),1.044 (95% CIs 1.002, 1.088; *p* = 0.039), 1.037 (95% CIs 0.908, 1.184; *p* = 0.594) and 1.062 (95% CIs 0.976, 1.156; *p* = 0.164). Overall, these results showed that both total IBD and CD could increase the risk of osteoporosis from a genetic perspective.

**Figure 5:**
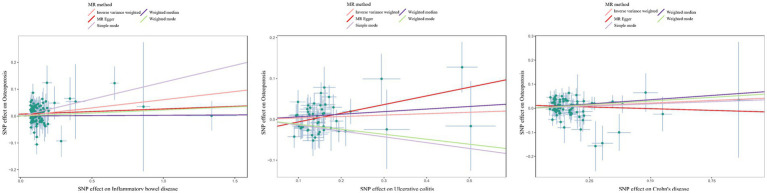
Scatter plot estimation of genetic risk for osteoporosis in the validation set IBD (including UC/CD). SNP: Single Nucleotide Polymorphism. The slope of each line represents the MR estimation effect of the different methods.

Although the scatter plot revealed that the total effect of IBD/CD/UC yielded a significant positive causal effect on the occurrence of osteoporosis, single SNP IVW analysis and leakage sensitivity analysis suggested that only IBD and CD yielded a positive causal effect on osteoporosis(Figure 5; Supplementary Figures S3, S4).

### Multivariate MR

To eliminate confounding factors related to UC and CD, we conducted a multivariate MR of UC and CD with osteoporosis to confirm our findings (26). Specifically, we identified SNPs associated with UC and CD separately (*p* < 5 × 10^−8^) and combined all genetic variants. Following the exclusion of duplicate IVs, we observed a significant causal association between CD and osteoporosis (*p* = 0.004) but no causal association for UC (*p* = 0.177). Our results were confirmed in the validation set, with a strong causal association between CD and osteoporosis (*p* = 0.040) and no significant association for UC (*p* = 0.755).

## Discussion

IBD is an immune-mediated idiopathic chronic digestive tract disease, often accompanied by various extraintestinal manifestations, such as erythema nodosum, bone and joint lesions, and ocular lesions (27). Epidemiological data suggest that individuals with IBD frequently exhibit reduced bone mineral density (with an incidence rate ranging from 30% to 80%) and are at a higher risk of developing osteoporosis (with an incidence rate of almost 50%). In severe cases, these patients may even experience fractures (28, 29).

The increased risk of fracture in patients with IBD, especially non-traumatic fractures, invariably poses a challenge to the management of this patient population. Due to the lack of a gold standard for diagnosing IBD, clinicians depend on a combination of clinical presentation, conventional diagnostic measures like endoscopy and histopathology, and eliminating other potential causes of colitis, infectious or otherwise. In cases of uncertainty regarding the diagnosis, endoscopic or histological evaluation should be conducted within a specific timeframe, typically within 6 months. DXA is widely used to determine BMD in patients because of its simplicity and non-invasiveness (30). The criteria for IBD patients eligible for DXA screening generally include postmenopausal status, ongoing or cumulative 3 months of corticosteroid therapy, history of fractures, and age over 60 years (31). However, these screening criteria are based on the bone physiology of postmenopausal Caucasian women, and their generalization to all ethnic groups still lacks adequate evidence. Additionally, IBD patients often have other risk factors for osteoporosis, and the small sample size of studies may overestimate its prevalence (29). BMD assessment can be quantified using T-score (as per WHO) and Z-score (as per ISCD) (32, 33). The T-score represents the number of standard deviations (SD) that BMD measurements deviate from peak bone mass in healthy individuals aged 30 years. Measurements within 1 SD of peak bone mass are considered normal, while measurements between 1 and 2.49 SD below peak bone mass indicate mild to moderate bone loss. Osteoporosis is defined by measurements that are more than 2.5 SD below peak bone mass (34). On the other hand, the Z-score is the number of SD that BMD measurements deviate from the mean BMD in healthy individuals of the same age group (35).

Various factors, such as daily diet, inflammation levels, low body mass index, advancing age, hypogonadism, reduced physical activity, and corticosteroid therapy, can all contribute to changes in bone structure and density in patients (36–40). When the gut is in a state of inflammation, the rate and volume of mineral loss are increased (41). An increasing body of evidence suggests that intestinal inflammatory cells such as macrophages regulate bone formation and bone resorption through the RANK/RANKL/OPG axis, further affecting bone density and increasing the possibility of osteoporosis (42–44). In a study of 122 patients with IBD, Tsironi et al. found that age (greater than 55 years) (OR 5.08, 95% CI 1.90–13.57, *p* = 0.001) and low body mass index were independent risk factors for osteoporosis (45). Hamdy et al. found that about 45% of patients who underwent ileectomy had osteopenia, and about 26% had osteoporosis (femoral neck and lumbar spine). Accordingly, ileal resection is a prognostic factor for osteoporosis (46). As an immunomodulator, vitamin D mediates various autoimmune diseases, including IBD (47, 48). Current evidence suggests that Vitamin D deficiency is more common in CD and UC patients than healthy individuals (49, 50). Interestingly, CD patients exhibit seasonal variations in vitamin D levels and bone turnover markers, and the long-term impact of such fluctuations on osteoporosis risk remains uncertain (51). In addition, the vitamin D receptor and its gene polymorphisms have attracted significant interest in recent years. In this regard, Bsml polymorphisms are closely related to osteoporosis (52). A survey study on the relationship between living habits and osteoporosis in Polish IBD patients found that the probability of osteoporosis in CD patients was 48.6%, while that in the UC group was 33.3%. Besides, CD patients diagnosed with osteoporosis were significantly less physically active (*p* = 0.0335) (29).

Moreover, the side effects of corticosteroids affect bone density and structure by inhibiting osteoblast activity and inducing apoptosis (53). There is an increasing consensus that BMD in IBD patients is correlated with the dose of corticosteroids administered (54, 55). Interestingly, some patients with IBD who received long-term steroid therapy did not experience significant bone loss. In contrast, at diagnosis, steroid-naive patients already had significantly lower lumbar spine BMD (56), suggesting that additional factors are involved in the pathophysiology of bone loss in IBD. As a result, BMD serves only as one of the risk factors for osteoporosis or fracture and may not always provide a reliable correlation measure. It is well-established that gene regulation plays a significant role in the heightened susceptibility to osteoporosis. At the level of gene transcription, low-density lipoprotein receptor 5 (LRP5) is involved in the transcription of human bone tissue and is thought to enhance bone resorption (57). When LRP5 undergoes deleterious mutations or loss of function, bone tissue develops phenotypic and functional deficits (58). Interestingly, osteoporotic pseudoglioma syndrome is a disease caused by loss-of-function mutations in the LRP5 genome, further supporting the critical role of this gene in bone integrity and function (59).

A prospective study across multiple countries suggested that about 12%–22% of IBD patients had asymptomatic vertebral fractures, including 19.6% (44/224) in Canada, 21.8% (34/156) in Germany, and 12.2% (22/179) in Israel (60–62). It remains unclear whether the reduction in BMD among CD patients is correlated with the occurrence of fractures or osteoporosis. In addition, the decrease in BMD in IBD patients often has no obvious phenotype, and the direct causal association with osteoporosis and fractures is unclear (63). D. Leslie et al. found that IBD only slightly increased the risk of BMD and osteoporosis (64). Univariate and multivariate analysis of CD patients by C. Noble et al. showed that low body mass index (BMI < 18.5) was associated with osteoporosis (*p* = 0.021, OR 5.83, CI 1.31–25.94) (65). Silva and colleagues conducted a study with 444 participants (case: control = 1:3) in Sri Lanka to investigate whether IBD is a risk factor for osteoporosis in adults. The team performed DXA bone density scans and found that while the overall incidence of osteoporosis was higher in the IBD group compared to the control group (13.5% vs. 4.5%, *p* = 0.001), there was no significant difference between UC and CD (14.45% vs. 10.7%, *p* > 0.05) (66). A prospective study on osteoporosis screening strategies in IBD patients found that nearly 66% of patients receiving steroids (>500 mg) were at risk of developing osteoporosis, including 77% for CD and 58% for UC (*p* < 0.001) (7). Therefore, the incidence of osteoporosis in IBD patients is also subject to confounding factors.

Previous research indicates that the prevalence of osteoporosis (as measured by T-score) in CD and UC is not significantly different (67, 68). However, after controlling for multiple confounding factors (age, hormone therapy, etc.), it was found that CD patients had an increased risk of osteoporosis by about 50% (T < −2.5), but this phenomenon was not observed in UC patients (68). Given that the mean reduction at each measurement point was less than 10%, the study could not assess the overall risk association between IBD and osteoporosis. Additionally, multiple studies acknowledge that CD activity has a more significant effect on BMD and bone physiology than UC. Haschka et al. suggested that, unlike UC, which has an acute onset and is limited to the distal colon, CD patients have a prolonged and systemic disease course and exhibit a more severe bone loss phenotype (69). In addition, CD is characterized by pathological changes in the small intestine, and inflammation or surgical resection of this area can lead to nutrient malabsorption and estrogen deficiency (70). However, Schoon et al. did not observe differences in BMD between CD and UC patients, and BMD was not significantly lower in newly diagnosed IBD patients (67).

Observational studies have indicated a possible direct causal link between IBD and a high prevalence of BMD loss and osteoporosis. However, there is currently limited emphasis placed on screening for osteoporosis in IBD patients. Additionally, due to the low absolute risk of fractures, there is considerable debate over whether all IBD patients should be screened. Therefore, excluding the interference of confounding factors, exploring whether there is a clear causal association between IBD and osteoporosis will benefit the preventive screening and specific treatment of osteoporosis. Genetic markers may assist in screening or identifying potentially high-risk patients for osteoporosis, and MR analysis based on GWAS can be used for causal analysis of IBD and osteoporosis.

Over the past few years, several variants involved in IBD/UC/CD development or immune-related biological processes have been reported, refining our understanding of the underlying pathological mechanisms. Among these, IL-1 and IL-18 are crucial in the inflammatory response and in maintaining intestinal mucosal stability. Nonetheless, their associations with the pathogenesis of IBD are not yet clearly understood. A recent study by Mi et al. revealed a positive causal effect of IL-18 on both IBD/CD/UC with odds ratios of 1.240, 1.199, and 1.274, respectively, in a three-sample MR study. In contrast, the genetically predicted IL-1 receptor antagonist (IL-1Ra) was negatively associated with the risk of IBD/CD/UC with odds ratios of 0.915, 0.902, and 0.899, respectively (71). This study suggests that genetically predicted IL-18 and IL-1Ra correlate with the risk of IBD, increasing the potential for drug development from a genetic perspective. Richards et al. coincidentally discovered three SNPs (rs17229943 (OCLN), rs385076 (NLRC4), and rs71478720 (IL-18)) that exhibited genome-wide significance for IL-18 levels using MR analysis (72). These three SNPs explained 6.8% of the variance in IL-18 levels, and the standard deviation of IL-18 under genetic prediction was positively associated with IBD susceptibility (OR = 1.22, 95% CI = 1.11–1.34, *p* = 6 × 10^−5^) (72). rs71478720 is located in the IL-18 intron and affects the expression of IL-18 in tissues, including the lung, thyroid, and skeletal muscle (73). rs385076 is an intron variant that maps to the NLRC4 locus and whose gene product, NLRC4 inflammatory vesicles, responds primarily to bacterial invasion (74). Activation of NLRC4 often induces the production of IL-1β and IL-18 and is also involved in cellular scorching (75). Its missense activating mutations often cause activation of auto-inflammatory disease, but the symptoms are distinctly tissue-specific (76). Besides, rs17229943 is an intron variant near the OCLN gene, primarily involved in encoding a membrane closure protein located at a tight junction (77). Buret et al. found that IL-18 could increase the inflammatory response to IBD by promoting neutrophil migration across the epithelium by selectively breaking the loop of tight junction closure proteins (78). It has been established that rs6062504 (related to TNFRSF6B) acts primarily as a mediator of the intestinal anti-inflammatory response to TNF-α (78). In addition, rs2488389 (associated with DENND1B) is expressed mainly in dendritic cells and natural killer cells and is involved in the synthesis of TNF receptor 1 (79). Moreover, rs2066845 (related to NOD2) is mainly involved in mucosal immunity and microbiota crosstalk (80, 81). Finally, it has been reported that rs11741861 (associated with IRGM) is primarily involved in intestinal mucosal autophagy and cytokine expression. The deletion of its upstream 20 kb copy number variant has been associated with increased or decreased IRGM in a cell lineage-dependent manner and is considered to be a possible causal variant (82).

Herein, we identified a causal association between CD and osteoporosis based on GWAS, enriching the existing framework for genetic variants that predispose to autoimmune disease. The strength of our study is its ability to overcome potential confounding factors present in observational studies, such as clinical heterogeneity arising from disease duration, sample quality, microbiota composition, clinical interventions, and diagnostic criteria. The large sample size ensures greater consistency in the obtained results, making it feasible to evaluate causal effects. However, the generalizability and applicability of the study’s findings to other populations are limited due to the exclusive use of a European sample base.

At present, multiple factors limit the widespread use of MR. Firstly, establishing a reliable genotype-intermediate phenotype-disease association can be challenging in the presence of ethnic or population diversity, which increases the risk of confounding among the three factors. Secondly, while GWAS has enhanced MR application, identifying suitable genetic variants as instrumental variables has become more intricate. Additionally, pleiotropy can introduce bias into MR results, especially if a genetic variant affects multiple phenotypic traits or has multiple biological effects (73). Thirdly, the inclusion of GWAS data from a larger population of middle-aged and older adults in MR studies may lead to amplification or splitting of gene effects due to cumulative environmental influences and interactions, potentially violating the MR assumption (71, 83). Excluding the potential impact of developmental adaptation during the interpretation of MR study results may pose a challenge (72). Finally, genetic variation may only explain a small proportion of exposures or traits in most cases (84).

Therefore, a large sample size is essential to obtain precise risk estimates when applying MR. Additionally, regression MR allows for a simplified assessment of the impact of exposure on outcomes through linear or logistic regression and facilitates the evaluation of effect sizes (85). Establishing a null outcome sample set is crucial for MR, as it links genetic variation and exposure rather than outcome, indicating that exposure does not affect the outcome. In the future, MR studies will aim to predict new biomarkers of disease onset (exposure) and screen for possible therapeutic targets by intersecting multi-omics data. The development of new algorithms represents a future direction to enhance the practicality of MR and minimize the aforementioned inherent limitations.

## Conclusion

The use of MR studies not only sheds new light on the causal relationship between inflammatory bowel disease and osteoporosis but also aids in generating new hypotheses regarding the development of osteoporosis. This progress lays the groundwork for future investment in validation intervention trials, exploration of novel therapeutic targets, and guidance for drug design. Although MR has limitations, conducting randomized investigations of biological mechanisms with the necessary genetic data, exposure, and outcome variables can better allocate resources and facilitate clinical trials. In summary, the extension of MR study methods minimizes the influence of confounding factors, strengthens epigenetic causal inference, and serves as a valuable reference for multidisciplinary disease treatment.

## Data availability statement

The original contributions presented in the study are included in the article/Supplementary material, further inquiries can be directed to the corresponding authors.

## Ethics statement

All datasets provided in GWAS have been approved by the relevant ethics committees.

## Author contributions

ZD, WX, and RD completed the construction of the model and the writing of the article. XP, XS, JS, and PD complete quality control, data analysis, and visualization. ZW and YL completes model guidance, critical review, and funding support. All authors contributed to the article and approved the submitted version.

## Funding

This work was supported by the National Natural Science Foundation of China (Nos. 82270549, 82000481, 82002507, and 82073056), the Shanghai Sailing Program (No. 20YF1429400), the Qingfeng Scientific Research Fund of the China Crohn’s & Colitis Foundation (CCCF) (No. CCCF-QF-2022C14-21), and Shanghai Pujiang Talent Program (19PJ1407600 and 20YF1430100).

## Conflict of interest

The authors declare that the research was conducted in the absence of any commercial or financial relationships that could be construed as a potential conflict of interest.

## Publisher’s note

All claims expressed in this article are solely those of the authors and do not necessarily represent those of their affiliated organizations, or those of the publisher, the editors and the reviewers. Any product that may be evaluated in this article, or claim that may be made by its manufacturer, is not guaranteed or endorsed by the publisher.

## Footnotes

^1^ https://doi.org/10.6084/m9.figshare.20603214.v1

## Abbreviations

BMD: bone mineral density
CD: Crohn’s disease
DXA: dual-energy X-ray bone absorptiometry
GWAS: genome-wide association studies
IBD: inflammatory bowel disease
ICD-10: International Classification of Diseases-10
IIBDGC: International Inflammatory Bowel Disease Genetics Consortium
IVW: Inverse variance weighted
LRP5: lipoprotein receptor 5
MR: Mendelian randomization
SD: standard deviations
UC: ulcerative colitis
UKIBDGC: United Kingdom IBD Genetics Consortium
