# Supplementary material for: Two-sample Mendelian randomization analysis evaluates causal associations between inflammatory bowel disease and osteoporosis
Source: Front Public Health. 2023 May 26;11:1151837. doi: 10.3389/fpubh.2023.1151837 (PMC10250718; doi:10.3389/fpubh.2023.1151837)
Supplement: Supplementary file 1 [file Table_1.DOCX]

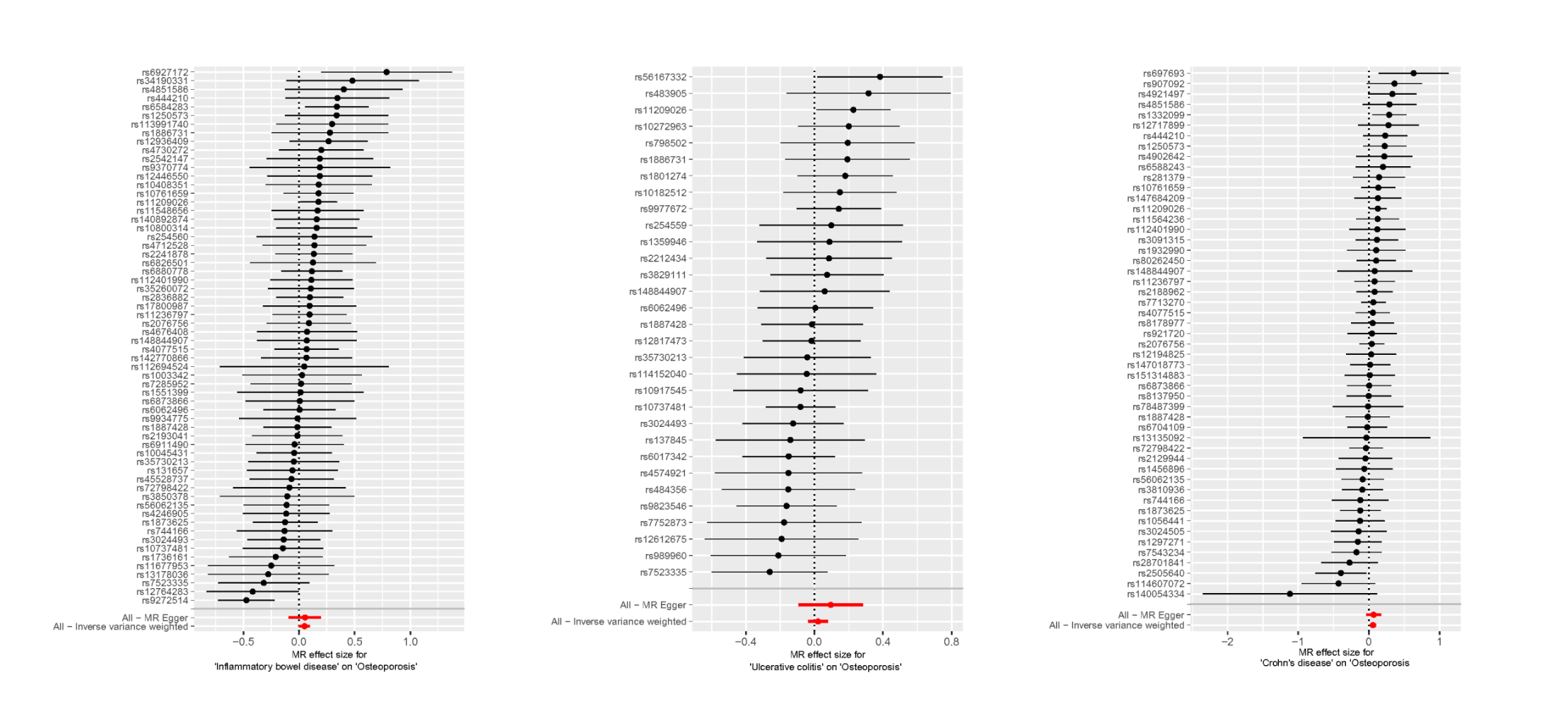


FigureS1: Single SNP IVW analysis assessed the causal association between IBD/CD/UC and osteoporosis in the training set.


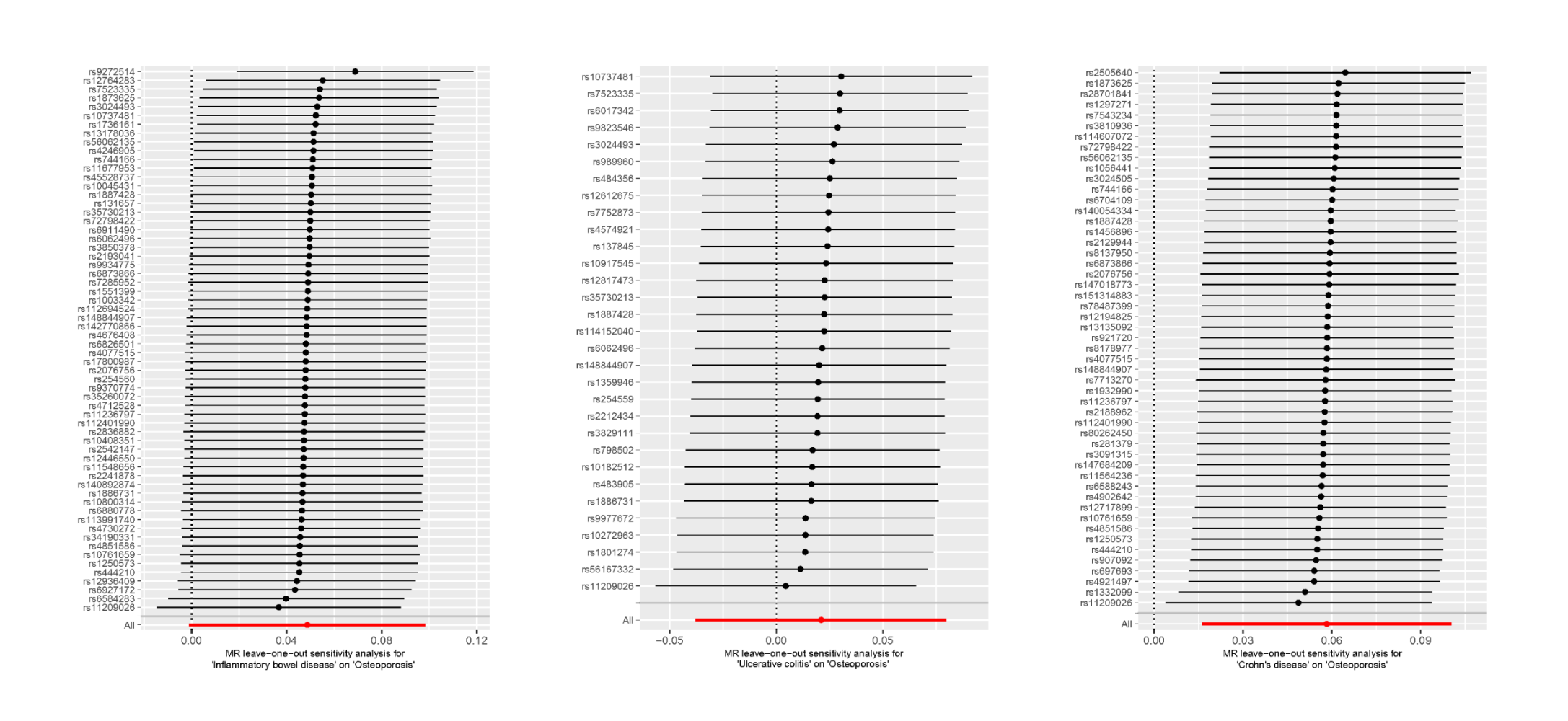


FigureS2: The leave-one-out sensitivity analysis assessed the causal association between IBD/CD/UC and osteoporosis in the training set.


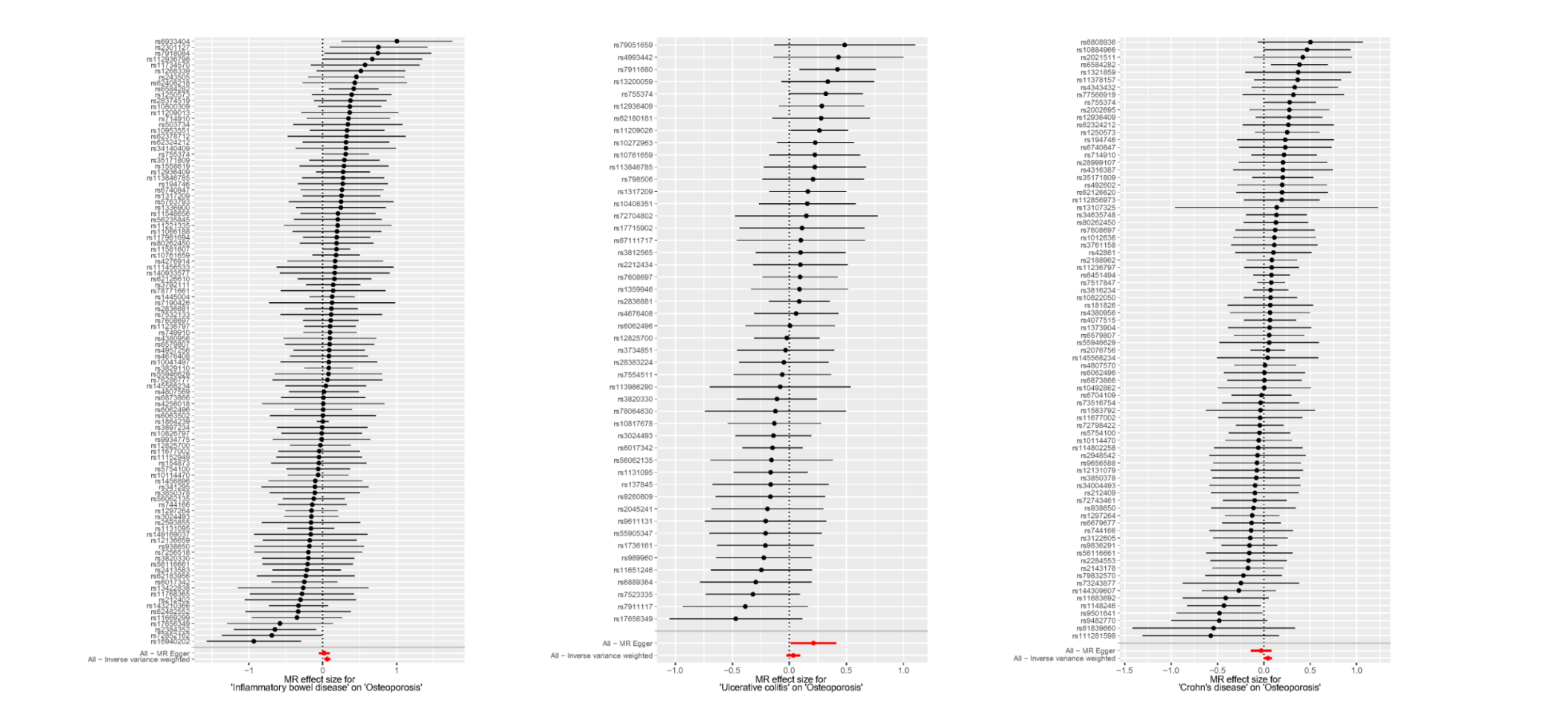


FigureS3: Single SNP IVW analysis assessed the causal association between IBD/CD/UC and osteoporosis in the validation set.


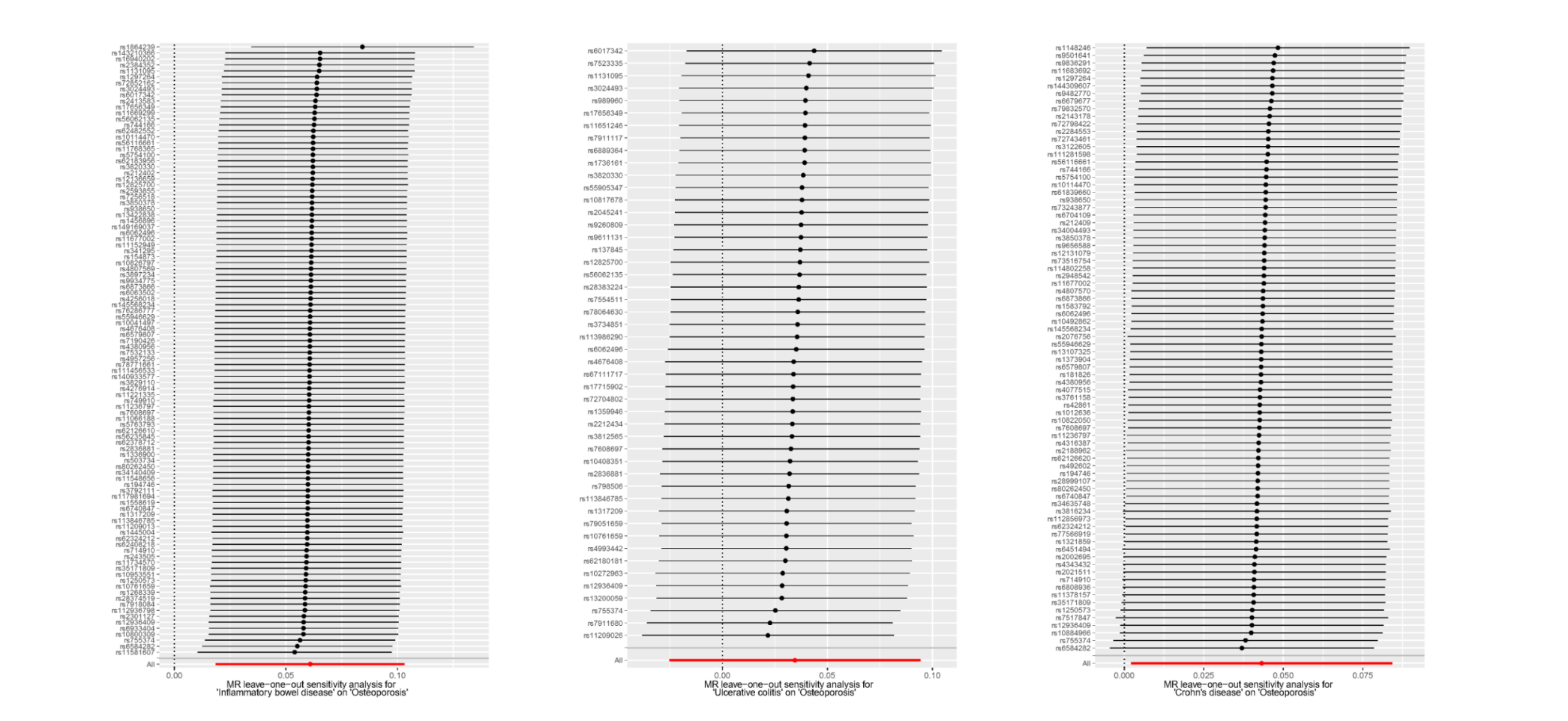


FigureS4: The leave-one-out sensitivity analysis assessed the causal association between IBD/CD/UC and osteoporosis in the training set.
